# Developing an online learning community for mental health professionals and service users: a discursive analysis

**DOI:** 10.1186/1472-6920-12-12

**Published:** 2012-03-21

**Authors:** Janet Smithson, Ray B Jones, Emily Ashurst

**Affiliations:** 1 School of Psychology, College of Life and Environmental Sciences, University of Exeter, Washington Singer Laboratories, Perry Road, Exeter EX4 4QG, UK; 2 Plymouth University, England, UK

## Abstract

**Background:**

There is increasing interest in online collaborative learning tools in health education, to reduce costs, and to offer alternative communication opportunities. Patients and students often have extensive experience of using the Internet for health information and support, and many health organisations are increasingly trying out online tools, while many healthcare professionals are unused to, and have reservations about, online interaction.

**Methods:**

We ran three week-long collaborative learning courses, in which 19 mental health professionals (MHPs) and 12 mental health service users (MHSUs) participated. Data were analysed using a discursive approach to consider the ways in which participants interacted, and how this contributed to the goal of online learning about using Internet technologies for mental health practice.

**Results:**

MHSUs and MHPs were able to discuss issues together, listening to the views of the other stakeholders. Discussions on synchronous format encouraged participation by service users while the MHPs showed a preference for an asynchronous format with longer, reasoned postings. Although participants regularly drew on their MHP or MHSU status in discussions, and participants typically drew on either a medical expert discourse or a "lived experience" discourse, there was a blurred boundary as participants shifted between these positions.

**Conclusions:**

The anonymous format was successful in that it produced a "co-constructed asymmetry" which permitted the MHPs and MHSUs to discuss issues online, listening to the views of other stakeholders. Although anonymity was essential for this course to 'work' at all, the recourse to expert or lay discourses demonstrates that it did not eliminate the hierarchies between teacher and learner, or MHP and MHSU. The mix of synchronous and asynchronous formats helped MHSUs to contribute. Moderators might best facilitate service user experience by responding within an experiential discourse rather than an academic one.

## Background

There is increasing interest in online collaborative learning tools in health education, to reduce costs, and to offer alternative communication opportunities for professionals, students and service users. Recent research findings have highlighted positive effects of e-health methods such as computerised cognitive behavioural therapy for reducing depression and anxiety [1], online forums for social support [2]. Implementation of new methods, and take-up by professionals, is slow [3]. It is sometimes argued that notions of 'teacher' and 'learner' are changing, through Internet technology use, towards collaborative learning between different stakeholders [4,5]. Many service users are experienced in social media, online forums and chatrooms, and many already use the Internet to find health information, get support, or "self-diagnose" [6]. Patients, especially those with long term conditions or ongoing health problems, can develop extensive knowledge from Internet sites or discussion forums. In this changing context, and with online services for mental health becoming more prevalent, there is a need for health care professionals to understand the possibilities for, impact of, and the limitations of, online technologies.

Meanwhile, there is increasing emphasis on service user involvement in mental health education [7,8]. Schneebli et al. [7] described a "philosophical shift from passive recipient of services to active participant at all levels of service delivery and policy development" between service users and mental health professionals, which has led to service user involvement as a "mandatory requirement of services", and increased calls for the involvement of mental health service users (MHSU) in the education of mental health professionals (MHP) [8]. However, despite this increasing focus on making medicine more "patient centred", a variety of studies of professional-patient interaction [9,10] have highlighted the persistence of professional dominance and the "remarkable persistence of asymmetry" [11]. For example, Marvel's [12] study of face-to-face interaction found that doctors regularly interrupt patients, not allowing them to complete their statement of concern. Pilnick and Dingwall [11] argued that "asymmetry lies at the heart of the medical enterprise: it is founded in what doctors are there for". They viewed this as not just a feature of medical profession dominance, which needs to be (or indeed can be) reduced by improved medical education, but an active co-construction of patient and professional, and concluded that "issues of asymmetry are key to understanding the ways in which these professionals and their patients organize their interactions' [11]. There is thus a tension between increased expectation of service user involvement, and of patient-centred care, and the expectations and behaviour of both professionals and service users in medical encounters.

These three changes: increasing patient use of the Internet for health purposes, online health service provision becoming more prevalent, and increasing emphasis on service user and health care professional interaction, have led to interest in collaborative online learning between healthcare professionals and service users-attempts to use the emerging technologies to develop new ways of learning and interacting. The intervention was motivated by the failure of previous projects to facilitate engagement from health care professionals in online discussions with service users. Reported barriers to involvement in previous studies include workload, lack of confidence and concerns relating to private-professional boundaries, duty of care and accountability, need for training, and limited experience [13-15]. In light of the shift towards involving service users in MHPs' education, the aim of this intervention was therefore to see if an alternative, more structured online "course" with a shorter time frame and more focused discussions would engage MHPs as well as MHSUs, and to try and produce a context in which both groups could discuss and learn from each other, away from the professional-patient service encounter.

Could an online format blur traditional teacher-learner boundaries and encourage new ways of relating and learning? MHPs and MHSUs are likely to have different expectations and norms for interaction and learning [4,16], and a detailed study of their online interactions and activities may aid development of appropriate learning and communication tools. There is evidence that new medical students are enthusiastic to participate in interactive learning [17], and so may be engaged with the concept of joining an online collaborative learning environment, or a "Community of Practice" [4,18].

In this article we address two main research questions:

1. How did MHPs and MHSUs interact on an online collaborative forum?

2. What helped or hindered collaborative learning in this online medical education context?

## Methods

### Course structure

The intervention comprised 3 week-long "courses", each with 12 discussion topics. Courses started with a 6 pm Wednesday live interactive webcast [19], a week of discussion forum, then a closing webcast the next Wednesday. The premise (from previous studies) was that having a scheduled event might help structure and encourage attendance.

### Participants

We recruited 19 MHPs and trainee MHPs (clinical psychologists, occupational therapists, mental health nurses) via online mailing lists and personal contacts from NHS trusts and higher education. We recruited 12 MHSUs via a previous project [13] and an independent user involvement service. Mean age was 42 for MHPs, 41 for MHSUs. There were more women: 13/19 MHPs and 10/12 MHSUs were women. Those wishing to participate completed an online registration form, gave consent by entering their email address, and provided an anonymous username for use during the course. Participants were followed up by email after the course.

### Moderators

The two researchers (authors 2 and 3 of this paper), who were not MHPs, took on moderation activity throughout the course-posting in response to questions, or if a post went unanswered.

### Procedure

Participants joined the website for an hour long interactive webcast. A MHP and the project lead presented a webcast, including a PowerPoint presentation of uses of the Internet for mental health, with intervals for typed instant message discussions. Discussion topics (Table 1) included lifestyle change websites, online therapies, use of Skype and email. Webcast topics and chat transcripts were posted to the forum for continued discussion throughout the week. On the final day, participants again joined a live interactive webcast with summaries of the week's discussions, eliciting further discussion and course feedback.

**Table 1:** Discussion topics

Discussion topics	
**1. CCBT/Online therapies**.	Does anyone have experience of using or recommending Living Life To The Full or Moodgym, or any other CCBT (computerised cognitive behaviour therapy)? Describe and discuss those experiences. If no experience in the group, then discuss whether you see a role for this, and why (perhaps) you have not used or recommended it up to now

**2. Discussion forums**	Do you think discussion forums are useful for people with mental health problems? Do any of you think that maybe, in some circumstances, a mental health professional should suggest signing up to a forum? ............. or is it just for that person to make their own decision?

**3. Lifestyle change intervention websites**	Given the evidence, and the limitations of the evidence, that you have heard so far, but taking into account the low marginal costs of such e-health interventions, do you think Internet based 'lifestyle changes' (diet, alcohol, smoking, sexual behaviour etc) programs can be useful? Might they have some role in mental health services?

**4. Webcast group therapy**	Practitioners: how would you feel about running a 'therapy' group webcast for a small group of your patients, where you were 'known' and in the video window, but they were anonymous? Mental health service users: how would you feel about taking part in such a session?

**5. Videophone**	Do you think Skype (Internet video calls) between patients and mental health professionals is worthwhile?

**6. Email**	What do you think about the use of email between patients and mental health professionals?

**7. Computer-patient interviewing**	Do you think computer-patient interviewing could play a role in mental health services

**8. Map of Medicine**	Do you think aids such as the Map of Medicine could play a role in mental health services?

**9. Patient access to their online medical records**	How would you feel about the sharing of online medical records between mental health professionals and patients?

**10. Barriers to Internet use in Mental Health practice**	What do you think prevents greater use of the Internet in mental health practice and how can it be overcome?

**11. Use of Internet for particular groups**	Are there particular groups of patients for whom the Internet would be most useful? And are there other groups for whom the Internet would not be useful? What are those groups and what are the differences?

**12. Implementation and requirements for supporting Internet uses**	Given what you have heard over the last week how would you like to see the Internet being used in mental health services? What would be needed to support these uses of the Internet?

### Ethics approval

The project was approved by the NHS South West REC (project 10/H0206/38).

### Analytic approach

The analysis uses a discursive approach, drawing on elements of discursive psychology (DP), and conversation analysis (CA) to understand how participants on a support forum use the forum and understand their and others' activities, Using this approach, language is understood to be a resource through which all sorts of interactional work can be accomplished [20,21], and analysis begins from what participants themselves are making relevant in their talk. What activities are participants doing in their online posts for advice or support, and what responses do their posts receive? The analysis begins from what participants made relevant in their talk, evidenced by what they oriented to in their posts, and in their responses to others. We pay particular attention to how professionals and service users interacted, how the online learning communities developed, and what helped or hindered collaborative online learning. There have been many studies of professional-patient interaction using discursive or conversation analysis [9-11]. Maynard and Heritage argue that "the co-constructive and collaborative analytic approach of CA emphasises the conduct of both parties" [10], and recently these methods have been adapted to study online health and mental health forums. Lamerichs and Te Molder [22], considered how participants managed 'the dilemma of support'-how to ask for support for a mental health problem, while simultaneously displaying 'competence' in managing their life. Vayreda and Antaki [23] explored advice-giving in a bipolar disorder online forum. Stommel & Koole [24] considered how people develop a sense of "belonging" to an online mental health forum.

While applying discursive and conversation analytical approaches to Internet data has limitations-for example, sequencing issues and timing of responses can be obscured, as the articles cited here demonstrate, many core aspects of a discursive approach can be used to understand forum talk. In an online context where people cannot use face-to-face interactional cues to determine the identity of a speaker, the ways in which forum posters use structural features of interaction, as well as content of their posts, become particularly important.

The data set for this analysis is the written posts in all three week-long courses, no software programmes were used for this as the quantity of text produced was manageable without this. All threads in all courses were read by all three authors, but the analysis presented here is primarily the analysis of the first author, who had not been involved in the data collection or moderation. The second and third authors, who had designed the course and moderated it, provided critiques and reflections on the analysis conducted. The data set (all the threads in the three courses) was scrutinized line by line in consideration of the two research questions of interest here: How did mental health professionals and service users interact on an online collaborative forum? Did the MHP's expertise discourage the MHSUs from posting? What appeared to help or hinder collaborative interaction from both parties?

The analysis in this paper is based on a few selected threads, and, in line with the majority of discursive analyses, is not intended to be representative, but rather seeks to focus on a theoretically and empirically interesting phenomenon, see for example [6,22,23]. In a discursive analysis, data is not "coded", but attention is paid to sequencing of posts, to exact words and phrases used, to timing of responses (where relevant), and to the fine details of exactly how someone performs the activity of posting, as evidenced by their written post, and the ways in which these details tend to elicit systematic responses. The extracts selected and presented here are chosen for their relevance in illustrating aspects of collaboration between MHPs and MHSUs, and how participants from both groups "used" the site. The phenomena described were all found recurring on other threads, and across the three courses. One participant read and provided feedback on the analysis.

## Results

### Creating a collaborative learning space

Participants come to an online space with expectations of relevance and how to post [25]. Here, the MHPs, MHSUs, and researchers started off with assumptions about relevant information and appropriate behaviour, which can be seen in what participants chose to introduce as relevant, and how these contributed to posting expectations. Previously, anonymity had been shown to be important to MHPs [13], who worried about professional implications of giving advice online. Anonymity was therefore central to this course design, and our aim had been to try and provide a non-hierarchical interactional space for MHPs and MHSUs. However, studies of online forums show that participants routinely demonstrate their status as members or experts [26,27]. When joining this course, what information did people provide about themselves?

In the opening webcast, participants were welcomed with music (Vivaldi) and chat about the weather, until the official opening by Ray:

Extract 1: Course 2, First webcast

1. **Ray**^1 ^so please do introduce yourselves

2. **Manon **ok.. well Hi.. I'm a mental health practitioner.. and I became involved in

3. mental health fifteen years ago after I experienced a bout of depression and had support

4. from the services back then.. so I'm happy to be thought of as both a service user and prac

5. **Emily **Hello! Welcome to the course. As you know I'm Emily and I'm the Ehealth

6. Facilitator

7. **Ray **all, say maybe something about your use of the Internet?

8. **Sharpeye **Hi there I'm Sharpeye but feeling peaky eyed today! A mental health practitioner

9. from Scotland. Use the Internet everyday and a couple of social networking sites.

10. **Mole **Im a MH user, i have previously used forums (although not much in the last 6

11. months)

12. and was involved with the Sharp talk project. i am on the Internet alot though (facebook,

13. skype, Twitter, uni work etc.)

How did participants introduce themselves? Manon, first to respond, mentioned her role as a MHP, and then her past experience as a MHSU. Then Emily, introduced herself by her role on the course. Ray elaborated on his open-ended suggestion of introducing oneself (line 1), with a prompt "so how have people used the Internet?" (line 7), tied to the course aim of anonymity, including the possibility of not disclosing their status as MHP or MHSU. Sharpeye also oriented first to her MHP role, and then to her Internet use. In this course (and in the others), MHPs posted first, and set expectations. While there was opportunity for not disclosing status as MHP, MHSU (or both), the first posters, the MHPs, raised this themselves, consistently, as a relevant part of their identity on this course. Moreover, their way of talking-the things mentioned, and their language-reflected their MHP identity. In mentioning her service user experience, Manon drew on a medical discourse, couched in the language of the service providers: "experienced a bout of depression", "had support from the services". Mole talked about her MH use (line 10), then her forum and technology experience. The minimal punctuation and casual use of capital letters is standard chat forum typing [28], but contrasts with the more formal posting of the two MHPs.

The researchers, Ray and Emily, tried to frame their questions neutrally, being interested whether anonymous online participation would overcome distinctions between "service provider" and "service user". Participants' positioning as MHP or MHSU may be expected during a round of introductions for this course, but it is notable that many participants, and especially MHPs, reiterated this throughout the course.

**Ragdoll **(C1)"however I am not a patient"

**Hero **(C2) "I wonder if as a MHP I am too used to careful wording of letters and find writing spontaneous text quite difficult"

Participants demonstrated that MHP/MHSU status was relevant, even though not explicitly requested. In this opening extract, we see both explicit orientation to a position, plus positioning by utilising medical expert or user experience discourses.

### Format and interaction

A regular conclusion from comparisons of synchronous and asynchronous discussions is that synchronous interactions are useful in establishing social bonds, "a greater sense of presence and generating spontaneity" [29], while asynchronous discussions are useful for learning- or task-oriented communications [30]. In this intervention we used a mixture of synchronous and asynchronous formats, which provides the opportunity to compare the two. There were slight differences in posting frequency by MHPs and MHSUs in webcast posts (synchronous) and discussion forum (asynchronous)-an overall average of 11 webcast posts by MHPs and 13 by users, and an average of 7 forum posts by MHPs and 5 by MHSUs. Our main focus is however on the *ways *people post in each format. Participants were asked to visit a website offering self-help for depression "Living Life to the Full" http://www.livinglifetothefull.org.uk and comment on their experiences of similar sites:

Extract 2: Course 1, Webcast (synchronous) discussion, CCBT (computerised cognitive behaviour therapy)

1. **Alpaca **Last time i tried cognative therapy it just made everything worse, to me being told

2. that i was'thinking about things wrong' just added to my negative feelings about myself.

3. **Astra **I have used mood gym but found the whole thing very frustrating! Because I found

4. the systm hrd to use, and I found it so impersonal

5. **Hawk **i'll have proper look later when there's more time

6. **Shell **shouldn't using a CCBT site only really be tried if CBT in general has been

7. recommended as a treatment

8. **Reflector **I think I might find the front page of Living Life to the Full quite text-heavy if

9. experiencing very low mood

10. **Hiker **Is that part of why the someone supporting is so important-because these sites

11. can feel impersonal?

12. **Astra **Shell: i was recomeded to use it, but i tink your right

13. **Reflector **Hi Hiker-yes I think support is vital for (1) guidance through the process and

14. (2) if a peer, understanding where you are coming from in a personal way

In this discussion, Alpaca and Astra described their experiences of and feelings about using therapy (real life, or online "mood gym"), and the effect on their mood, thereby providing user perspectives, or 'experiential" accounts of the therapies. Shell subsequently argued that a therapy should be recommended. In contrast to the experiential responses, Reflector and Hiker posted more formally, conventional punctuation, using medical expertise or language, e.g. "if experiencing very low mood" (line 9). Astra (Line 12) responded directly to Shell. Reflector's second post (lines 13-14) is a direct response to Hiker's question with numbered points.

### "Listening" to other participants

On a webcast it can be unclear whether posts are in direct response to the original question, or in response to a later post, but participants indicate this in various ways. Some referred directly to others' posts by name (lines 12, 13), but also by "mirroring" the terms used-Hiker (line 11) repeated Astra's word "impersonal" (line 4), and Astra (line 12) repeated Shell's word "recommended". The "thread" of the discussion was thus picked up, in a fast synchronous format, participants demonstrated they were responding to other posts, not just directly to the original question-an indication that, as hoped, MHPs and MHSUs were "listening to" each other.

### Moderator intervention

The above extracts were from the synchronous webcast. Extract 3 is the related asynchronous discussion over the following week.

Extract 3: Course 1, asynchronous discussion, CCBT

1. **Emily Wed 17:00**: Does anyone have experience of using or recommending Living Life To

2. The Full or Moodgym, or any other CCBT? Describe and discuss those experiences. If no

3. experience in the group, then discuss whether you see a role for this, and why (perhaps) you

4. have not used or recommended it up to now

5. **Alpaca Wed 22:00: **Personally i haven't used them up until now because i didn't know that

6. they existed. It seems like a good concept, but from what people have said earlier, doesn't

7. apply to everyone in a positive way.

8. **Puff Thu 9:14: **I have used Mood gym a little but as I said I found it a little awkward to use.

9. It is also about what your state of mind at that time as well, how positive you are when you

10. start. I think there is a place for them though and as things develop, surely they will just get

11. better?!

12. **Ray Thu 9:45: **Puff I was wondering if you can remember in what way was it awkward?

13. You probably know that MoodGym is Australian in origin, whereas LivingLifeToTheFull is

14. British. I don't know if that makes any difference at all. Both have been'tested' in various

15. randomised trials and NICE http://guidance.nice.org.uk/TA97 recommends them as one

16. possibility for people with depression (long description of results of trials here)

17. **Emily Thu 13:34**: Hello, here's a summary of last night's second discussion:

18.-LLTTF-Text-heavy content can be off-putting, some content can add to negativity.

19.-MoodGym-Can be frustrating, awkward to use as impersonal and formulaic.

20.-A mixture of experiences in using Computerised Cognitive Behavioural Therapy.

21.-Severity of condition and present state can affect CCBT usefulness.

22.-Suggestions that CCBT should only be used if personally suitable type of therapy and on-

23. to-one support to monitor if it's helpful or not.

24. Some willing to try particularly if personal therapy waiting lists are long.

25. **Hiker Thu 20:03: **I'm going to have a look at them. Haven't used them up to now because I

26. see them as aimed at mild to moderate and thats not my field. If I used them it would be to

27. do it alongside someone.

28. The evidence for CBT is always hard to compare with other approaches. My gut feeling is

29. that used alone it doesn't make lasting changes. I'd expect the same here.

30. I feel I'm being very negative here but I don't mean to be, I think I'm just very cautious about

31. all these things. Do other people feel that way too?

32. **Puff Thu 21:53: **It was the keeping up the exercises bit. I am trying to remember why i

33. particularly found it awkward it was quite a while ago (but maybe for me its more that i

34. have a bad view of CBT in general). maybe using a few online methods together, like mood

35. gym with a skype session follow up or skype preceding it, Make it a bit more human. you

36. dont want to feel'palmed off' onto the internet, like your not worth bothering with.. there

37. needs to be some good explanation to its purposes/uses and support whilst on it..

38. **Ray Tue 16:08**: Given the comments by some of you about CCBT not being for you, I

39. thought I would have another look at the literature on acceptability and drop out from using

40. CCBT. There is a study which is a review of other papers, entitled The acceptability to

41. patients of computerized cognitive behaviour therapy for depression: a systematic review

42. (Psychological Medicine (2008), 38, 1521-1530.).

43. Here is the summary of their study: (long summary follows)

(No more posts on this thread).

Emily started with a question intended to suit both MHSUs and MHPs "used or recommended". This elicited two MHSU responses, Alpaca and Mole, drawing on their user experience. Ray responded to Puff, referring to NICE guidelines and medical trials. Emily then posted a summary of the synchronous webcast (Extract 2). Hiker (MHP) posted, responding to the "evidence", expressing her reservations, and finished with an open question, "do other people feel that way too?" (line 31). Puff returned and appeared (line 32) to answer Ray's question (line 12) about the way therapy made her feel. Nothing further was posted for 5 days, then Ray posted another CCBT literature summary, which was not responded to before the course closed the next day.

### Researcher, MHP and MHSU expectations

We can note different expectations participants brought to the course. The researchers wanted to generate an online collaborative learning space, and acted as social hosts, "weaving" the threads [5], while posting to open up the discussion and minimise hierarchies between participants. MHPs signed up wanting training, and to receive a certificate. MHSUs offered and compared "experiential knowledge". Did the MHPs orient to the MHSU's experiential knowledge? In each course, some topics attracted more posts from the MHPs (e.g. "would online therapy work?", "Useful websites"), topics which encouraged providing information and considering research. Other topics attracted more posts from MHSUs ("sharing patient records online", "barriers to Internet use"); who typically responded, as Astra and Alpaca did, from an experiential perspective. The researchers introduced the 'researcher' view, with literature summaries and links, in contrast to moderation on other sites which focused on supporting the MHSUs [13]. The researcher intervention here was specifically concerned with learning and discussion, rather than support.

Extract 4: Course 3, asynchronous discussion: Who would benefit from online therapies?

1. **Emily Wed 15:39 **Are there particular groups of patients for whom the Internet would be

2. most useful? And are there other groups for whom the Internet would not be useful? What

3. are those groups and what are the differences?

4. **Tranquility Sat 19:25**: I'm thinking there are at least two useful groups-1 being those who

5. frequently use the Internet and who naturally turn to the Internet for information/

6. communication-Younger and middle aged people (HYPERLINK).

7. I think the second group would be those most likely to be advantaged by use-people with

8. social phobias, agoraphobia, those who work unsocial hours, people in rural locations,

9. people with disabilities or caring responsibilities. Perhaps the second group who might have

10. the most to gain?

11. **Toucan Sun 18:37: **I don't disagree with you Tranquility, but I do often question whether

12. the Internet is useful for those with social phobia/agoraphobia. I haven't come to the answer

13. myself yet, but I do wonder whether the potential of providing a less anxiety provoking way

14. of interacting with others is a double edged sword. As well as putting the individual in

15. contact with others, it could also reinforce avoidant behaviour. I suppose it comes back to

16. something I mentioned in the chat during the webcast-is our definition of social contact

17. changing?

Emily again posted an open question without explicit reference to status. There was a three day gap before the first reply; Tranquility categorised "two useful groups", with a supporting link. As with Reflector in extract 2, this post was framed as a considered academic argument. One day later, Toucan replied, starting with "I don't disagree with you Tranquility, but....", a formulation understood as a careful management of disagreement [31]. Again the medical expert/academic discourse is noticeable, Tranquility and Toucan drew on professional ways of talking-"reinforce avoidant behaviour" (line 15). In this asynchronous discussion, there was more emphasis on the evidence, the professional view, and less of the experiential perspective.

### Perceived proportion of MHPs and MHSUs

A primary motivation for this intervention had been the limitations of previous studies in engaging MHPs in online learning in a self-harm support forum. This led to the "course" format with a participation certificate, efforts to recruit more MHPs, and focused discussions. In course 3 (Extract 5) there were more MHPs (7:3).

Extract 5: Course 3, first webcast

1. **Toucan **- I'm not convinced that Internet use does reduce social contact, I think it

2. challenges our tradition concept of what can constitute social

3. **Toucan **- tradition-al even!

4. **Lex-**i think it easy to be drawn into other people's problems and feel sad by them and

5. have no outlet for them

6. **Manon **- but that's the problem, Toucan.. I have observed people loosing the

7. confidence in social situations with actual people, whilst feeling fine in the world of their

8. chat sites...

9. **Milkyway **- i worry as there are some dodgy ppl about

10. **Milkyway **- mental health sufferers can be very vulnerable

11. **Manon **- so what might go wrong, Milkyway? that you might get to know someone

12. who's dodgy...?

13. **Toucan **- Yes Lex, I agree with you. I think that when using discussion forums you can

14. find yourself in a role of not being able to leave a discussion or getting trapped into a helper

15. role. I have certainly experienced this issue with a service user

The positioning of MHSUs as the other, "very vulnerable" (line 10) is notable here. In this extract MHSU/MHP status is not immediately clear, but posters use of medicalised or academic discourses ("it challenges our traditional conception"), including formal ways of describing a problem "I have observed people losing their confidence in social situations"-again, positioning these people with problems as other people, observed not experienced. One of the few MHSUs on this course, Hawk, commented early on that "I feel outnumbered". The self-correction of grammatical inaccuracy (line 3) is a feature of formal academic discourse, in contrast to forum speak [28]. Again we see how posters indicate they are "listening" and responding to others, with explicit references (lines 6, 11, 13) to others by name. The final extract comes from Course 1, in which 2 MHPs were outnumbered by 9 MHSUs.

Extract 6: Course 1, final webcast

1. Shell as a user i would have preferred a higher ratio of users:professionals

2. Ray Shell, yes the next one will have many more professionals. That was partly down to us

3. being rather late in getting the adverts out to professionals.

4. Shell @ray-no, i want MORE users

Shell's perception of many professionals is interesting, given the proportions on his course. Our analysis suggests several explanations for a perception of more MHPs, even in course 1 when they were in a minority, and though there were as many posts overall by MHSUs. MHPs tended to post first, setting interactional norms of using a medical or academic discourse. There was overlap of MHP/MHSU roles-several MHPs mentioned prior MHSU experience, and some MHSUs worked in health professions. Moreover, many MHSUs are veteran forum users, which may give them "contributory expertise" [32] in their MH area.

## Discussion

### Format and discussion style

We were interested in if and how professionals and service users interacted in this format, and whether it provided space for collaborative learning. Previous research has demonstrated the need for different modes of online course for groups with differing needs [33]. The slower asynchronous format elicited more MHP posts, and elaborate, information-based discussions. Explicit indication of learning is more obvious here, but the synchronous mode may have an important role in encouraging MHSU participation, and experiential posts. In both formats there were indications of posters "listening", as evidenced by their explicit references to and responses to previous posts. This was a key research aim, and may contribute to improved patient-professional understanding [34]. Extra synchronous webcasts-perhaps an extra midweek one-might help increase MHSU involvement.

### Engagement, hierarchy, and status

In terms of the project's initial aim to try and provide a non-hierarchical space for MHPS and MHSUs to interact, there was clear evidence of participants posting in ways which reproduced the MHP/MHSU distinctions. MHPs were highly visible in these online courses, posting first, setting site norms. For future collaborative learning, it is useful to recognise different MHSU and MHP motivations. MHPs had the clear objective of improving their technical knowledge, MHSUs used the site to discuss lived experience. In contrast to previous projects [13], this approach worked in getting the two groups talking online. We conclude that as a general format the structured anonymous course works in getting MHSUs and MHPs together, and the mix needs to ensure that neither group feels'outnumbered'. Given the difference in confidence in posting on these topics, on a shared forum, having more MHSUs may provide a perception of equal groups.

### Medical, academic and experiential expertise

Kerr et al. [35] noted how in public science debates, technical expertise tends to be privileged over lived experience or "experiential expertise". Here too, more academic/medicalised discourses were more responded to, which may have contributed to marginalisation of MHSU positions [36]. While there was overlap in participant categories (many posters had both MHP and MHSU experience), and little age difference between the two groups, there was a distinct contrast between use of academic/medical expert discourses, often drawn on by the MHPs, and the experiential discourse drawn on by MHSUs, who also used less formal language, in line with MH forum norms [37]. Despite the "flatter" hierarchy, participants' tendency to position themselves as MHP expert or as a lived experience service user was pervasive. However, interactional asymmetry and distinctive stakeholder roles are not necessarily a problem [10], the intention was to provide a context in which MHPs and MHSUs could talk and listen, which our analysis here suggests occurred. Besides the analysis of the online talk, the participants provided written feedback after the course, analysed in a separate publication [38], in which both MHSUs and MHPs reported satisfaction with their involvement, and most of the MHPs said they now had plans to implement e-health measures into their practice, subject to external limitations such as trust policy.

### Moderation

The moderation style, in keeping with the intervention's aim as a collaborative learning course, was within the academic/medical discourse, with references to literature and research findings, rather than orienting to the MHSU's feelings. While the moderators tried to phrase posts carefully to encourage neutral, open-ended opportunities to discuss topics, this analysis highlights the impossibility of moderator "neutrality" [39,40]. From this analysis, we suggest that it would be useful to include moderators with "lived experience" of being or having been MHSUs, and/or attention be paid to responding to the MHSUs in "experiential" mode.

## Limitations

This was a small pilot study looking at the feasibility of "user-led online learning" in three small groups. The three short courses can only provide indications of what worked and what did not work, and what needs further investigation. The MHPs recruited were interested in learning about the internet, so MHPs who resist Internet technology were not involved in this study, and the findings cannot be assumed relevant for MHPs in general. Similarly the MHSUs were self-selected and already familiar with Internet use, and already prepared to interact with MHPs in this course.

## Conclusions

In this article we considered two main research questions. Firstly, how did mental health professionals and service users interact on an online collaborative forum? This analysis suggests that the collaborative format was successful in as far as MHSUs and MHPs were able to discuss issues together, listening to the views of the other stakeholders. The format achieved was not, however, anonymous or non-hierarchical in terms of participants' background and training: participants in this "anonymous" space constantly positioned themselves in lay and expert roles. Despite this asymmetry between lay and expert discourses, this structured format allowed both MHPs and MHSUs to feel sufficiently comfortable to post, in a "co-constructed asymmetry".

Secondly, what helped or hindered collaborative learning in this online medical education context? Our study suggests several possible facilitators to online collaborative learning which may help in developing online learning environments:

• Providing a mixture of formats encourages participation by a range of stakeholders. A synchronous format may encourage service users to describe their perspectives, and an asynchronous format may elicit reasoned debate. Specifically, for this course an extra webcast midweek may have facilitated MHSU involvement.

• The proportion of professionals to service users is important. This analysis suggests that to give an impression of similarly weighted groups, it may be worth recruiting fewer MHPs than MHSUs.

• The course format provided a place for MHPs and MHSU to interact but moderators may need to ensure that stakeholders feel comfortable posting, an academic discourse may inhibit service user involvement.

### Suggestions for future research

This pilot study involved a small group of self-selected MHPs and MHSUs. Future research could include MHPs who are resistant to online technology. It would be useful to include moderators from both academic and lived experience groups in future research, as this may facilitate responsiveness to different MHSU and MHP styles and needs.

## Abbreviations

CBT: Cognitive behaviour therapy; CCBT: Computerised cognitive behaviour therapy; MHP: Mental health professional; MHSU: Mental health service user.

## Competing interests

The authors declare that they have no competing interests.

## Authors' contributions

JS conducted the majority of the analysis and writing of this paper. RJ was the principal investigator and designer of the project and contributed to this analysis. EA was involved in conducting the research and provided some of the analysis. All authors read and approved the final manuscript.

## Endnotes

^1^Usernames have been changed, except for researchers, to provide "double anonymity".

## Pre-publication history

The pre-publication history for this paper can be accessed here:

http://www.biomedcentral.com/1472-6920/12/12/prepub
